# *Aspergillus fumigatus* is responsible for inflammation in a murine model of chronic obstructive pulmonary disease exacerbation

**DOI:** 10.1186/s12931-024-03092-7

**Published:** 2025-01-18

**Authors:** Alexandra Bouyssi, Alexis Trecourt, Tanguy Déméautis, Florence Persat, Olivier Glehen, Martine Wallon, Gilles Devouassoux, Abderrazzak Bentaher, Jean Menotti

**Affiliations:** 1 UR3738 CICLY Team Inflammation and Immunity of the Respiratory Epithelium, Claude Bernard University, Lyon 1, Pierre-Bénite, France; 2 Department of Pathology, South Lyon Hospital, Hospices Civils de Lyon, Pierre-Bénite, France; 3 Department of Medical Mycology and Parasitology, Institute of Infectious Agents, Croix-Rousse Hospital, Hospices Civils de Lyon, Lyon, France; 4 Surgical Department, UR3738 CICLY, Claude Bernard University – Lyon 1, South Lyon Hospital, Hospices Civils de Lyon, Pierre-Bénite, France; 5 Department of Pulmonology, Croix-Rousse Hospital, Hospices Civils de Lyon, and F-CRIN INSERM Network CRISALIS, Lyon, France

**Keywords:** Chronic obstructive pulmonary disease, Cigarette smoke, Exacerbation, *Aspergillus fumigatus*, Inflammation, Murine model, Macrophages, Epithelial cells

## Abstract

**Background:**

In patients with chronic obstructive pulmonary disease (COPD), a sensitization to *A. fumigatus* has been related to a decline in lung function, but the role of fungal agents in the disease pathogenesis remains unclear. The main purpose of the present study was to investigate whether cell inflammation could worsen after exposure to *A. fumigatus* spores in vitro and then, in mice, following chronic exposure to cigarette smoke mimicking COPD.

**Methods:**

The inflammatory response to cigarette smoke alone or with *A. fumigatus* was investigated in cell culture models of murine macrophages and alveolar epithelial cells. In an animal model, mice were exposed daily to two cigarettes smoke over 14 weeks, and two intranasal instillations of 10^5^ spores at weeks 7 and 14. Then, their lungs were recovered to perform inflammatory and histopathological analyses.

**Results:**

In co-cultures of macrophages and epithelial cells treated with both cigarette smoke extracts (CSE) and *A. fumigatus* compared to CSE alone there were significant inductions in *TNF-α* (6.2-fold) and *CXCL-2* (21.5-fold) gene expression, confirmed by significant increases in the corresponding protein secretion. In the murine model, histological analyses of the lung after chronic smoke exposure showed an increase in airspace enlargement. Moreover, a Bio-Plex approach on bronchoalveolar lavage of cigarette smoke and *A. fumigatus*-treated mice showed significant increases in multiple inflammatory proteins secreted in the lung.

**Conclusions:**

There was a stronger inflammatory response after cigarette smoke exposure with *A. fumigatus* compared to cigarette smoke alone. These findings were correlated with histopathological changes in the mouse lung in vivo.

**Supplementary Information:**

The online version contains supplementary material available at 10.1186/s12931-024-03092-7.

## Introduction

Chronic obstructive pulmonary disease (COPD) represents the third leading cause of death worldwide [1]. The most important cause of COPD is environmental whereby cigarette smoking plays a critical role [2]. Tobacco smoking-induced COPD corresponds to a chronic altered inflammatory response resulting in lung parenchyma destruction and emphysema development [3]. Cigarette smoke (CS) contains numerous toxic products that alter various immunological functions. For example, there is a marked recruitment of alveolar macrophages but their phagocytic function and cytokine production are disrupted; likewise, the ability of epithelial cells to respond to infection is also altered [4]. Conversely, abnormally activated immune cells secrete inflammatory mediators that will directly or indirectly breakdown lung structural architecture, impacting its physiological function [5].

COPD exacerbations, characterized by dyspnea and/or cough, are associated to cardio-vascular comorbidity and their frequency is associated with an accelerated lung function decline and high mortality rate [2, 6]. Exacerbations are primarily due to infections by bacteria, viruses, or fungi such as *Aspergillus fumigatus*.

*A. fumigatus* is a saprophytic airborne fungal pathogen, that may cause diseases such as allergic bronchopulmonary aspergillosis (ABPA), aspergilloma or chronic pulmonary aspergillosis in pathological lungs [7], and invasive pulmonary aspergillosis (IPA) in immunocompromised individuals [8].

An association between exposure to *Aspergillus* spores and symptoms associated with airway inflammation has been found [9, 10]. In COPD patients, *Aspergillus* is also found in a third of respiratory samples [11] and *A. fumigatus* sensitization has been associated with frequent exacerbations, deterioration of lung function and poor clinical prognosis [12, 13]. Fungal colonization or infection has been reported to be associated with COPD exacerbations, but the role of fungal agents in the COPD pathogenesis as well as in exacerbations remains unclear and is still debated [13, 14].

The objective of the present study was to investigate whether COPD could worsen after exposure to *A. fumigatus* spores (AFsp). To this end, mono- and co-cell culture models of both murine alveolar epithelial cells and macrophages, were exposed to cigarette smoke extract (CSE) and/or CSE and AFsp to identify their role in inflammatory responses. We then established a mouse model of chronic cigarette smoke (CS) exposure with or without two spaced instillations of AFsp to investigate in vivo lung inflammation and tissue changes.

## Materials and methods

### CSE preparation

The smoke of 2 3R4F cigarettes from the Tobacco Research Institute (University of Kentucky, Lexington, KY, US) was bubbled in 1 mL of PBS (100%).

### Aspergillus strain

The CBS144.89 strain of *A. fumigatus* was used for spore preparation as previously described [15].

### Cell culture

For mono-culture models, murine RAW 264.7 macrophages and MLE-15 lung epithelial cells were cultured in Dulbecco’s modified Eagle medium (DMEM) with 10% fetal bovine serum (FBS) and 1% penicillin/streptomycin at 37 °C in 5% CO_2_. Sub-confluent cells were treated with CSE at 5% (v/v) in DMEM, or DMEM alone, for 1 h and then with DMEM and AFsp at a multiplicity of infection (MOI) of 3 for 4 h (CSE + AFsp), or DMEM alone. Three independent experiments were performed.

For the co-culture model, MLE-15 were placed at first and, the following day, RAW 264.7 were added at a final ratio of 1 RAW 264.7 to 2 MLE-15 in a 12-well plate [16]. Sub-confluent cells were treated in the same way as the mono-culture models. Four independent experiments were achieved.

### Animal experiment – CS model

Twenty-three (12 male and 11 female, 7-week-old) Specific and Opportunistic Pathogen-Free (SOPF) C57BL/6J mice purchased from Charles River Laboratories (Saint-Germain-Nuelles, France) were used. Mice were housed in ventilated racks with 12 h light/dark cycle and water/food *ad libitum.* One female mouse died from hydrocephalus before the experiment began and 3 males died during the smoking period.

After an acclimation period of 1 week, mice were randomly divided into three groups: (i) a control group exposed to filtered air only with two intranasal instillations of PBS at week 7 and 14, (ii) a group exposed to the smoke of 2 (3R4F) cigarettes a day for 5 days a week over 14 weeks to mimic COPD (chronical exposure), and (iii) a group exposed to the smoke of 2 cigarettes a day for 5 days a week over 14 weeks and 2 intranasal instillations of 10^5^ AFsp at week 7 and 14, to mimic COPD exacerbation. Mice were placed in a specially designed smoking chamber and exposed to CS as previously described [17]. Mice were briefly anesthetized with 3% isoflurane before spore instillation. Mice were sacrificed 24 h after the last instillation. Then, bronchoalveolar lavage (BAL) fluids and blood were recovered for inflammatory mediator secretion analysis, as previously described [17, 18], and lungs were recovered to investigate inflammatory mediator gene expression and to perform histopathological analysis as well as *Aspergillus* DNA extraction.

All animal experiments adhered to the European Union Directive 2010/63/EU on the protection of animals used for scientific purposes. The protocol was approved by the French ministry of higher education and research after agreement from the local ethics committee (CECCAPP, approval number LS-2017-016).

### Quantification of *Aspergillus* DNA

DNA extraction from the lungs was performed using a Maxwell^®^16 CSC automaton (Promega, Madison, WI, US) after bead beating using a MagNA Lyser instrument (Roche, Basel, Switzerland). Then, an *A. fumigatus*-specific real-time PCR was performed in duplicate as previously described [19, 20]. For quantification, a standard curve was established between the threshold cycle (Ct) values and the spore numbers; this standard curve was used to determine the number of conidia by interpolation of the Ct value obtained by real-time PCR [19].

### Histopathological analyses

Lungs were fixed in formalin (Laurypath, Brignais, France), embedded in paraffin (Tissue-TEK Paraffin Wax TEKK III; Sakura, Osaka, Japan), cut into 3-µm thick tissue sections and stained with Hematoxylin-Eosin-Saffron (HES, Tissue-Tek Prisma & Glas G2; Sakura) and Grocott (Slide stainer; Ventana Medical System, Tucson, AZ, US). Tissue inflammation was observed and quantified, as previously described [16].

### Morphometry

Pulmonary tissue destruction was estimated by calculating the mean linear intercept (Lm), which corresponds to a measure of the airspace enlargement, as previously described [18]. Briefly, 20 randomly chosen fields from scanned pathology slides of each mouse’s HES-stained pulmonary tissue were recovered in a blinded manner, using the software ImageJ version 1.54d (NIH, Bethesda, MD, US).

### RNA extraction and cytokine gene expression

RNA extraction was performed using TRI Reagent (Sigma-Aldrich^®^, St Louis, MO, US) and Nucleospin RNA for NucleoZOL (Macherey-Nagel, Hœrdt, France), according to the manufacturers’ protocol. To exclude residual genomic DNA, the DNA-free kit (Thermo-Fisher, Waltham, MA, US) was used. RNA was reverse transcribed using the High-Capacity cDNA Reverse transcription kit (Thermo-Fisher). The cDNA obtained was used as templates for qPCR using TaqMan^®^ probes (Thermo-Fisher). Assays were carried out on an AriaMx Real-Time PCR thermocycler (Agilent, Vénissieux, France), using the 2^-ΔΔCt^ method [21] to determine the relative expression of genes. The gene coding for hypoxanthine-guanine phosphoribosyltransferase (HPRT) was used as housekeeping gene.

### Enzyme-linked immunosorbent assay (ELISA)

CXCL-1 and CXCL-2 were quantified in the supernatants recovered from co-cultured cell experiments using the Quantikine^®^ ELISA CXCL-1 and CXCL-2 kits (Biotechne^®^, Minneapolis, USA), and IL-1α as well as TNF-α using the ELISA MAX™ Deluxe Set IL-1α and TNF-α (BioLegend^®^, San Diego, USA), according to the manufacturer’s protocols; CXCL-2 was quantified in the BAL of mice using the same kit, and IgE using the ELISA MAX™ Deluxe Set IgE (BioLegend^®^).

### Multiplex assays of cytokines in BAL fluids and sera

Cytokine secretion in BAL fluids or sera was quantified using Bio-Plex™ Pro Mouse Cytokine 23-Plex Assay (Bio-Rad™, Marnes-la-Coquette, France), according to the manufacturer’s protocol. BAL were undiluted and sera were diluted 1:4.

### Statistics

All statistical tests were performed using Prism version 8.4.2 software (GraphPad Software, La Jolla, CA, US) and data were expressed as mean and standard deviation (SD) or median and interquartile range (IQR). Statistical tests were used to investigate differences in ΔCt for RT-qPCR and concentrations (pg/mL) for ELISA and Bio-Plex assays. To compare the differences between 3 groups, normality was verified using the Shapiro-Wilk test and then, either ordinary one-way ANOVA with Tukey’s multiple comparisons test, Kruskal-Wallis’ test with Dunn’s multiple comparisons test or Brown-Forsythe and Welch’s ANOVA with Dunnett’s multiple comparisons test were performed. A p-value < 0.05 was considered statistically significant.

## Results

### Cell culture studies

**Fig. 1:**
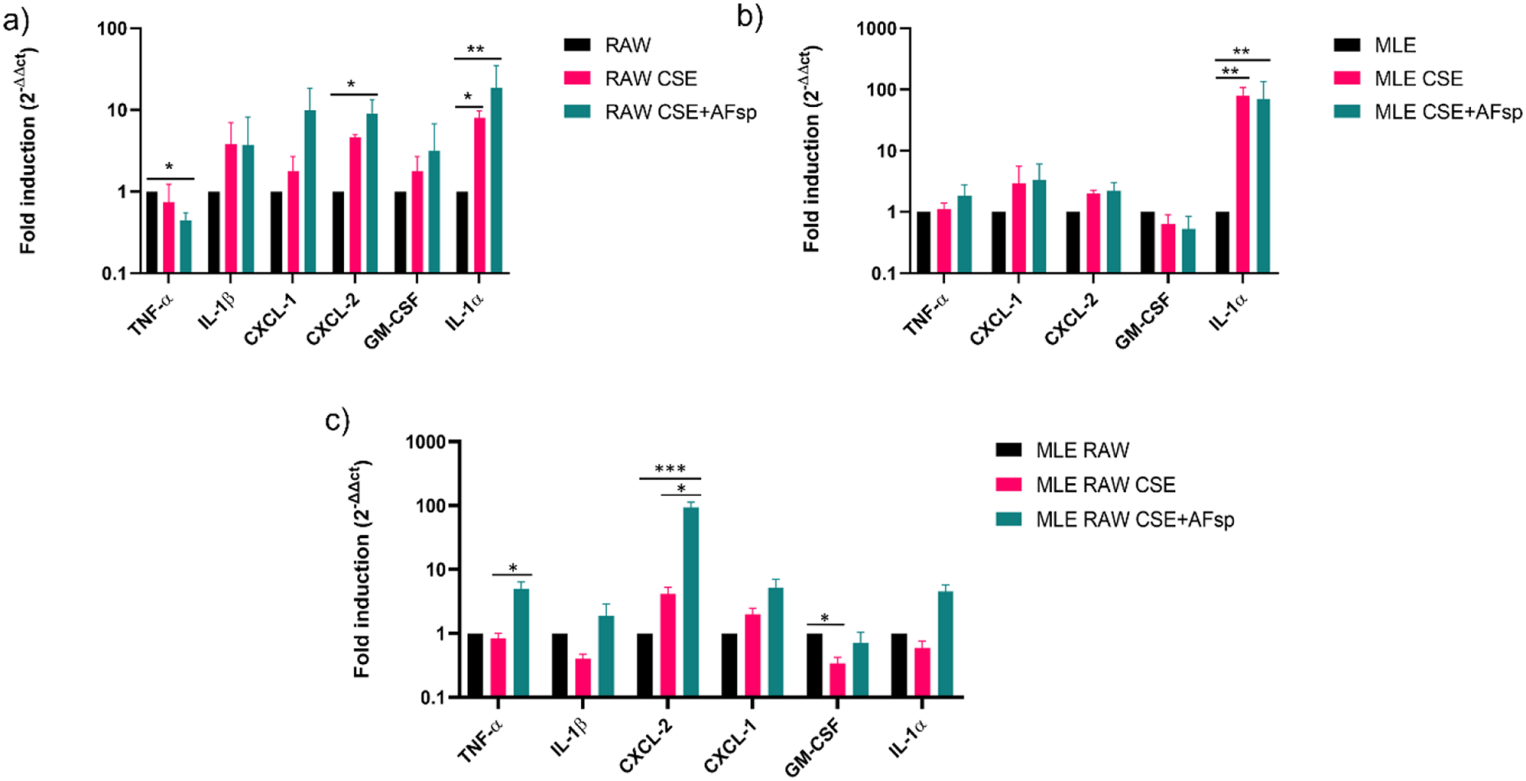
Gene expression of multiple mediators by RAW 264.7 and MLE-15 cells. Cells were exposed during 1 h to cigarette smoke extract 5% (CSE) and during 4 h to *A. fumigatus* spores at MOI3 (CSE + AFsp). (**a**) Fold induction of the gene expression of inflammatory mediators in RAW 264.7 cells. (**b**) Fold induction of the gene expression of inflammatory mediators in MLE-15 cells (**c**) Fold induction of the gene expression of inflammatory mediators in co-cultured cells. The mean ± SD is presented, *n* = 3 in monocultures and *n* = 4 in co-cultures, *: p < 0.05, **: p < 0.01, ***: p < 0.001

To investigate whether exposure to AFsp could worsen cell inflammation in an acute model, we first evaluated the gene expression pattern of several cytokines in RAW 264.7 macrophages and MLE-15 epithelial cells after exposure to CSE and to both CSE and AFsp. In RAW 264.7 exposed to CSE, compared to controls, there was an 8-fold significant increase in *IL-1α* expression (*p* = 0.0237), and a trend towards an increased expression of *CXCL-2* (4.6-fold, *p* = 0.0741). In MLE-15 exposed to CSE, compared to controls, there was an 88-fold significant increase in *IL-1α* expression (*p* = 0.0013). In RAW 264.7 exposed to CSE + AFsp, compared to controls, there was a 9-fold significant increase in *CXCL-2* expression (*p* = 0.02) and an 18.8-fold significant increase in *IL-1α* expression (*p* = 0.0073). Surprisingly, compared to controls, there was a 2-fold significant decrease in *TNF-α* expression (*p* = 0.0219; Fig. 1a). In MLE-15 exposed to CSE + AFsp, compared to controls, *IL-1α* expression was significantly induced 66-fold (*p* = 0.0027; Fig. 1b). In both RAW 264.7 or MLE-15 exposed to CSE + AFsp, compared to CSE alone, there was no significant difference in the expression of the mediators studied (Fig. 1a-b). Monocultures of murine macrophages and alveolar epithelial cells thus showed the increase in certain inflammatory cytokines in CSE- and CSE + AFsp-treated cells; however, as monocultures cannot reflect the inflammatory response due to the cooperation between different cell types and as there was no significant worsening of the inflammatory response after exposure to AFsp in CSE-treated cells, we then used a co-culture model of MLE-15 and RAW 264.7 to allow cross-talk between macrophages and epithelial cells, and more closely reflect physiological conditions.

In the co-culture exposed to CSE, compared to controls, there was a 3.7-fold significantly decreased expression of *GM-CSF* (*p* = 0.0161). However, in the co-culture treated by CSE + AFsp, compared to controls, there was a 99-fold significant increase in *CXCL-2* expression (*p* = 0.0010). Interestingly, in co-cultures exposed to CSE + AFsp, compared to those exposed to CSE alone, there was a 6.2-fold significant increase in *TNF-α* expression (*p* = 0.0243) and a 21.5-fold significant increase in *CXCL-2* expression (*p* = 0.0493; Fig. 1c). In co-cultures of murine macrophages and alveolar epithelial cells, inductions of certain cytokine gene expression were thus evidenced, which is compatible with our hypothesis that AFsp exposure worsen the inflammatory response induced by CSE exposure. We therefore wanted to investigate this response at the protein level.

**Fig. 2:**
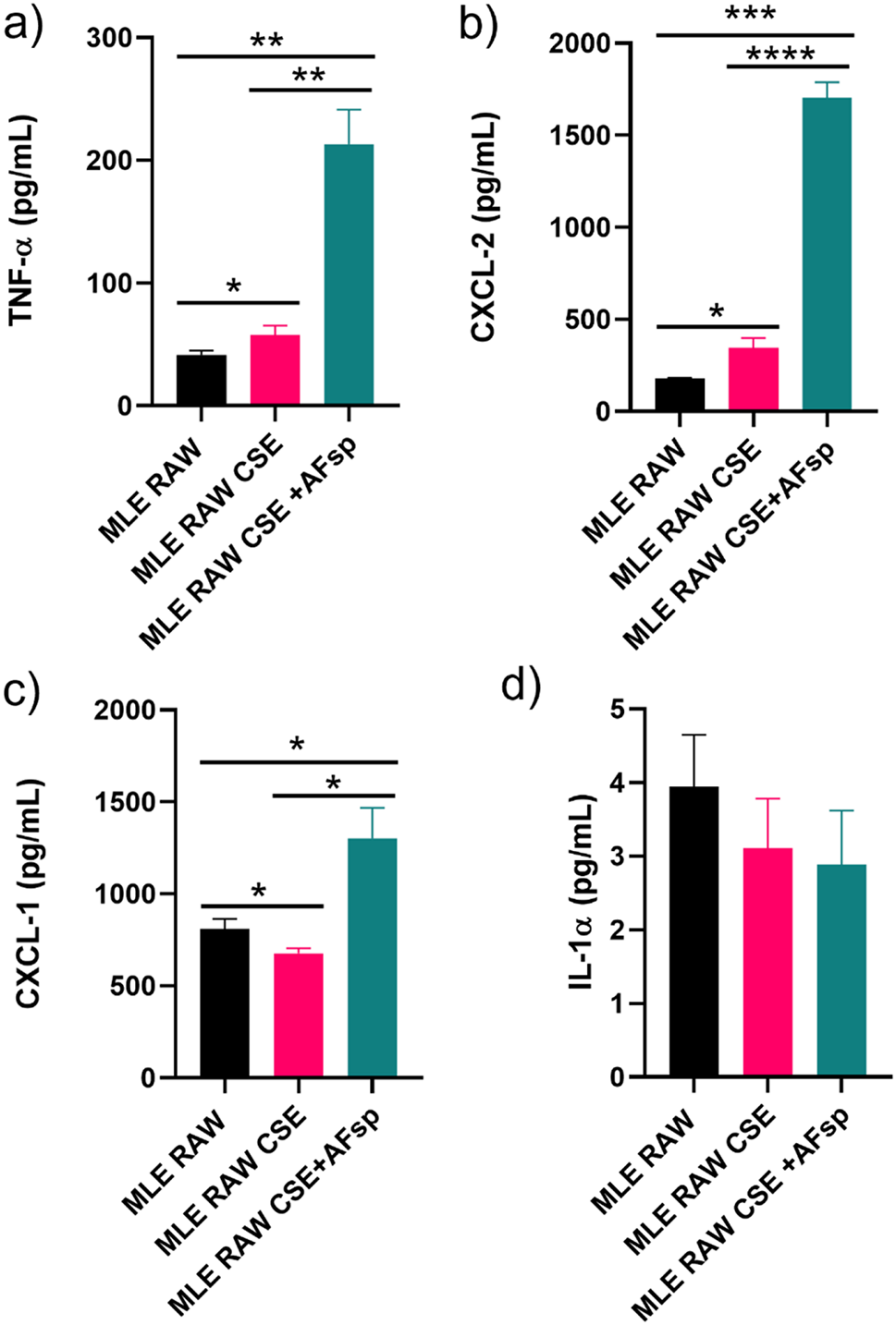
Quantification of mediators in the supernatants of co-cultured MLE-15/RAW264.7 cells exposed to CSE and CSE + AFsp: (**a**) TNF-α, (**b**) CXCL-2, (**c**) CXCL-1 and (**d**) IL-1α. The mean ± SD is presented, *n* = 4, *: p < 0.05, **: p < 0.01, ***: p < 0.001, ****: p < 0.0001

We next performed ELISA on 4 mediators to investigate whether the protein levels in the supernatant of co-cultured cells confirmed the mRNA expression profile. In co-cultured MLE-15 and RAW 264.7 exposed to CSE, compared to controls, there was 1.4-fold significant increase in secreted TNF-α (*p* = 0.0495; Fig. 2a) and a 1.9-fold significant increase in secreted CXCL-2 (*p* = 0.0193: Fig. 2b), while there was a 1.2-fold decrease in CXCL-1 secretion (*p* = 0.0348; Fig. 2c). In co-cultured cells exposed to CSE + AFsp, compared to controls, there was a 5-fold significant increase in secreted TNF-α (*p* = 0.0029; Fig. 2a), a 9.5-fold significant increase in CXCL-2 secretion (*p* = 0.0001; Fig. 2b), and a 1.6-fold significant increase in CXCL-1 secretion (*p* = 0.0123; Fig. 2c). In co-cultures exposed to CSE + AFsp, compared to CSE alone, there was a 3.7-fold significant increase in TNF-α secretion (*p* = 0.0043), a 5-fold significant increase in CXCL-2 secretion (*p* < 0.0001), and a 1.9-fold significant increase in CXCL-1 secretion (*p* = 0.0119). In co-cultures exposed to CSE alone as well as CSE + AFsp there was no significant difference in the secretion of IL-1α (Fig. 2d).

Taken together, these data indicate that, in co-cultures of murine macrophages and alveolar epithelial cells, exposure to AFsp worsened cell inflammation. We therefore further investigated these observations in an animal model of COPD exacerbation mediated by *A. fumigatus*.

### Animal studies

**Fig. 3:**
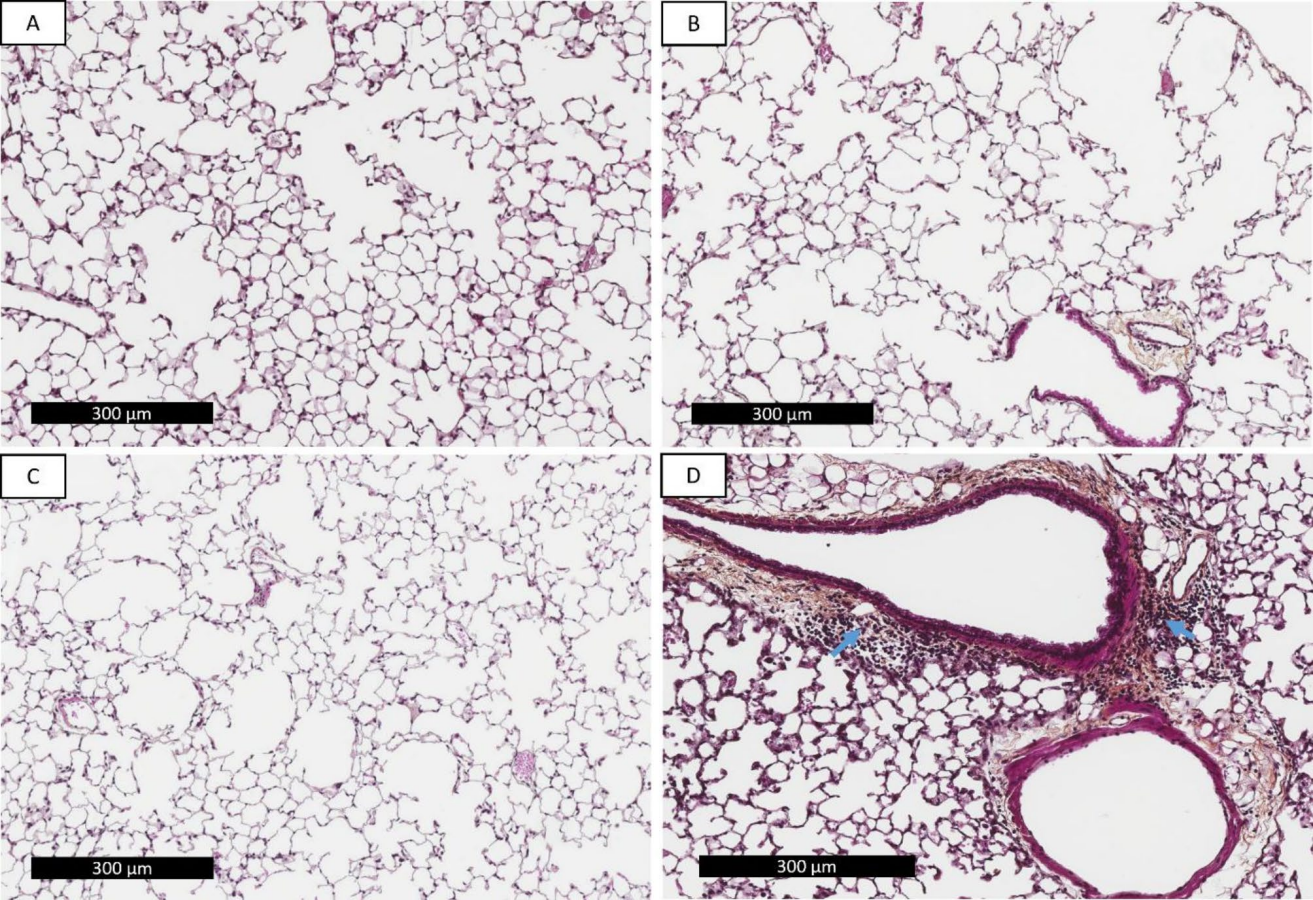
Tissue degradation and inflammation observed in formalin-fixed paraffin-embedded lung tissues. Representative images 10X (scale bar 300 μm) stained with hematoxylin, eosin, saffron (HES). **a**) Control mice exposed to filtered air, **b**) Cigarette-smoke (CS)-exposed mice, **c**) Cigarette-smoke and *Aspergillus fumigatus* spores (CS + AFsp)-exposed mice, **d**) inflammation in CS + AFsp mice – inflammatory cells are indicated by blue arrows. No fibrosis nor fungal elements were detected on the HES (or Grocott-stained slides). CS induced a notable airspace enlargement in both CS and CS + AFsp groups

**Fig. 4:**
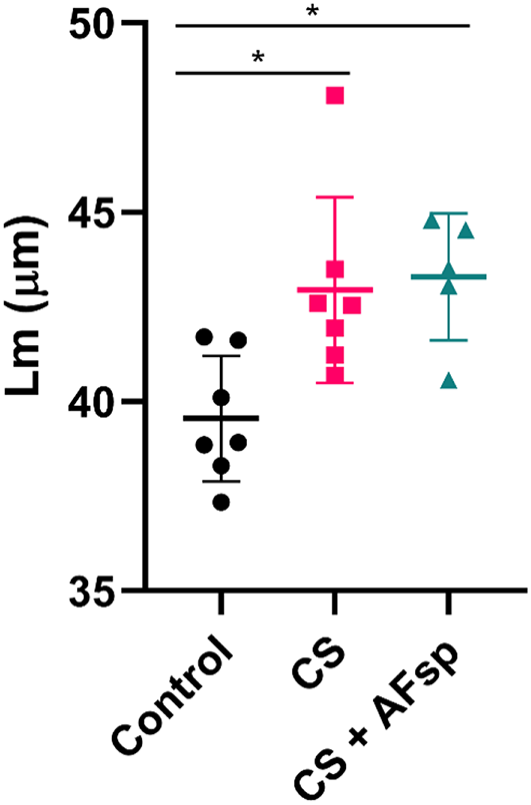
Marked airspace enlargement in both CS-exposed mice. Significant increases of the mean linear intercept (Lm) were observed in CS and CS + AFsp-exposed mice. The mean ± SD is presented, *n* = 5–7 mice per group, *: p < 0.05

During the smoking period, although they gained less weight (Supplementary figure S1a and b), mice showed no signs of stress. Exposure to CS led to perivascular inflammation in only 1/7 mouse, but bronchiolitis and presence of a perivascular edema in 6/7 mice. Moreover, CS induced a notable airspace enlargement in both CS-exposed and CS + AFsp-exposed mice compared to controls (Fig. 3a-c). This was confirmed by measurement of Lm as there was a significant increase both in CS (*p* = 0.0463, 1.1-fold) and CS + AFsp (*p* = 0.0137, 1.1-fold) groups compared to the control group; there was no significant difference between CS and CS + AFsp groups (Fig. 4). In the CS + AFsp group, inflammatory reaction and perivascular edema (Fig. 3d) were observed in all mice. A summary of pathological findings is presented in Supplementary Table S1.

**Fig. 5:**
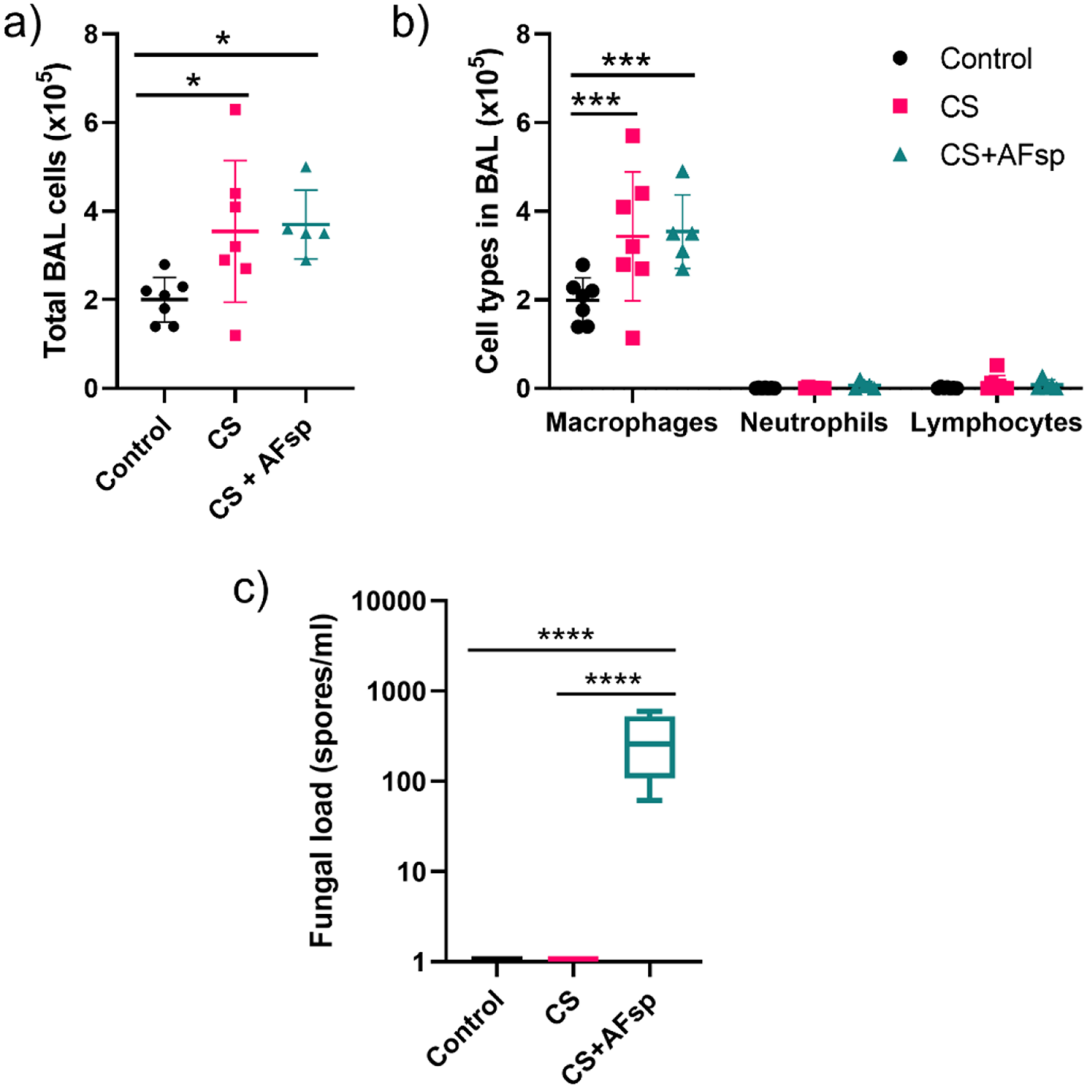
Assessment of cell count in BAL and fungal load in lung tissue. **a**) Total BAL cell count, **b**) cellularity in BAL and c) fungal load in lung tissue. The total cell count was performed with an automatic cell counter with trypan blue solution. Cell types were determined with cytospin slides. Data are represented by mean ± SD for **a**) and **b**). Box and whiskers indicate the median, IQR, as well as minimal and maximal values for **c**), *n* = 5–7 mice per group, *: p < 0.05, ***: p < 0.001, ****: p < 0.0001

To understand the underlying basis of tissue destruction and inflammation, we examined the inflammatory cell recruitment in lungs of mice. Compared to control mice, a greater total BAL cell count was observed in CS (*p* = 0.0458) and CS + AFsp (*p* = 0.0445; Fig. 5a); most cells were macrophages, with rare neutrophils and lymphocytes in cytospin (Fig. 5b). There was no significant difference between CS-exposed and CS + AFsp-exposed mice.

Lungs of control, CS and CS + AFsp-exposed mice were tested for the presence of *A. fumigatus* using specific qPCR. In control and CS mice, as expected, no fungal DNA was observed. In mice exposed to CS + AFsp, the median [IQR] fungal load was 260.9 [107.2-528.1] spore-equivalents/mL (Fig. 5c).

**Fig. 6:**
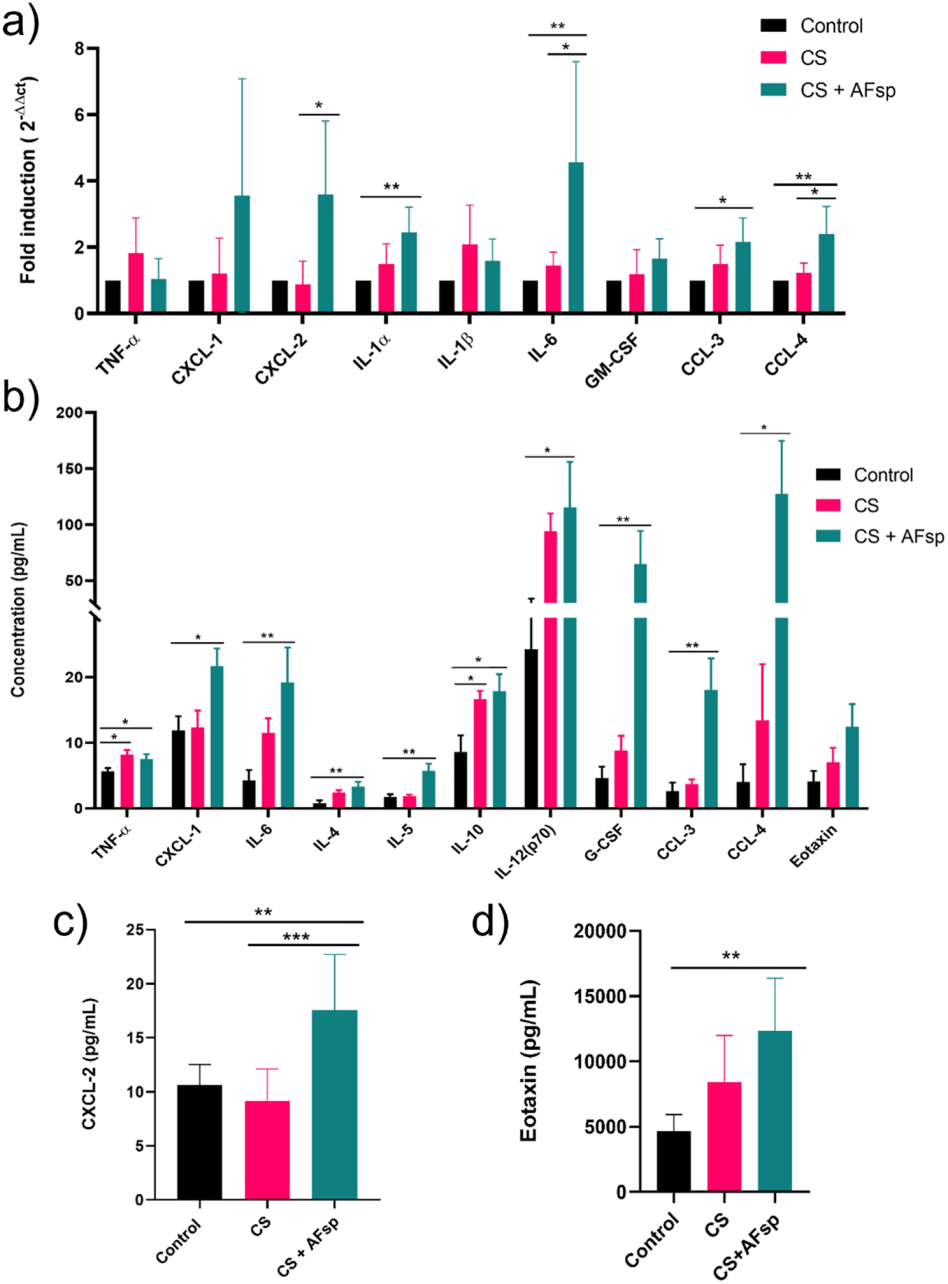
Gene expression and secretion of mediators in specimens from exposed mice. **a**) gene expression of inflammatory mediators in pulmonary tissue, **b**) concentrations (pg/mL) of some secreted cytokines in bronchoalveolar lavage (BAL) fluids, (**c**) CXCL-2 concentrations in BAL and (**d**) eotaxin concentrations in sera of mice exposed to cigarette smoke (CS) and CS + AFsp. Data are represented by mean ± SD, *n* = 5–7 mice per group, *: p < 0.05, **: p < 0.01, ***: p < 0.001

We next assessed the gene expression of some inflammatory markers to investigate whether these results would confirm the in vitro and the histopathological findings. In mice exposed to CS, compared to controls, there was no significant difference but there was a trend towards increased *IL-1β* expression (2.1-fold, *p* = 0.0869). In mice exposed to CS + AFsp, compared to controls, there was a 2.5-fold significant increase in *IL-1α* expression (*p* = 0.0072), a 4.5-fold increase in *IL-6* (*p* = 0.0046), a 2.2-fold increase in *CCL-3* (*p* = 0.0195) and a 2.4-fold increase in *CCL-4* (*p* = 0.0025); compared to CS, there was a 4-fold significant increase in *CXCL-2* (*p* = 0.0267), a 3-fold increase in *IL-6* (*p* = 0.0374) and a 2-fold increase in *CCL-4* (*p* = 0.0162) gene expressions were increased in CS + AFsp group, as well as a trend towards an increase in *CXCL-1* gene expression (3-fold, *p* = 0.0659; Fig. 6a).

To investigate the inflammatory markers involved in the CS response with or without *A. fumigatus* spores, we studied biomarkers in BAL fluids using multiplex immunoassay and ELISA. In mice exposed to CS, compared to controls, there was a 1.4-fold significant increase in TNF-α secretion (*p* = 0.0336) and a 2-fold significant increase in IL-10 (*p* = 0.0328; Fig. 6b). Additionally, there was a trend towards an increase in IL-4 that was 3-fold higher (*p* = 0.0594) and also IL-12(p70) that was 3.8-fold higher (*p* = 0.0719). In mice exposed to CS and AFsp, 11 proteins were significantly more secreted in the BAL compared to control mice (Supplementary Table S2), and there was a trend towards an increase in eotaxin that was 3-fold higher (*p* = 0.0622). In mice exposed to both CS + AFsp, compared to mice exposed to CS, there was a significant 2.2-fold increase in CXCL-2 secretion (*p* = 0.0003; Fig. 6c). Furthermore, there was a trend towards an increase in the secretion of IL-5 that was 3-fold higher (*p* = 0.0626) as well as CXCL-1 that was 1.7-fold higher (*p* = 0.0517; Fig. 6b).

To explore systemic inflammation, we performed an immunoassay on sera from CS, CS + AFsp and control mice. In sera of CS-exposed mice, compared to control mice, there was a trend towards an increase in eotaxin that was 1.8-fold higher (*p* = 0.0825). Eotaxin was also the only inflammatory mediator that displayed a significant increase in sera of CS + AFsp mice compared to controls (2.7-fold, *p* = 0.0015; Fig. 6d).

## Discussion

To explore whether COPD could be exacerbated after exposure to *A. fumigatus* spores, we developed acutely injured cell models with a single exposure to CSE with or without a single exposure to AFsp in mono- and co-cultures of murine RAW 264.7 macrophages and MLE-15 epithelial cells, the first sentinel cells involved in immune response. This was further investigated in a mouse model of chronic (daily) exposure to CS over 14 weeks with or without 2 AFsp intranasal instillations 7 weeks apart to mimic COPD exacerbations with sensitization to *A. fumigatus*.

We evaluated the acute inflammatory response to CSE and to CSE + AFsp of both cell types in mono- and co-cultures that allow a cross-talk between these cells and approximate the in vivo conditions. To our knowledge, no study has previously been performed to analyze inflammatory response to CS and AFsp co-exposure in a co-culture cell model. In our cell culture models, we evidenced inductions of *CXCL-2* and *IL-1α* gene expressions in CSE + AFsp-treated macrophages compared to untreated cells and only an induction of *IL-1α* expression for CSE-treated macrophages. Only *IL-1α* gene expression was induced by both CSE and CSE + AFsp-treated epithelial cells. Surprisingly, there was no induction of cytokine gene expression in monocultures cells treated with CSE + AFsp with respect to CSE alone-exposed cells, contrary to the co-cultures treated by CSE + AFsp, where *TNF-α* and *CXCL-2* gene expression was induced with respect to CSE-treated co-cultures. In the supernatants of CSE + AFsp-treated co-cultured cells, the concentration of TNF-α, CXCL-2 and CXCL-1 were higher than in CSE-treated cells and controls. These mediators are involved in the recruitment of neutrophils [22, 23], key cells involved in the development of COPD particularly its exacerbation and clearance of *Aspergillus* from the lung [24, 25]. In our acute co-culture cell model, exposure to AFsp after exposure to CSE resulted in a greater inflammation response compared to CSE. Taken together, these findings suggest a pathological role for AFsp in COPD exacerbation, able to induce an inflammatory cascade.

To explore the pulmonary and systemic inflammatory response to AFsp after chronic exposure to CS, we used a murine model with daily exposure to CS to mimic COPD and 2 exposures of 10^5^ spores 7 weeks apart to mimic COPD exacerbation mediated by *A. fumigatus.* Mice chronically exposed to CS alone displayed a marked airspace enlargement, as well as a greater recruitment of immune cells. No inflammatory mediator gene expression inductions were observed, in accordance with the previously described alteration of the ability of alveolar macrophages from CS-exposed mice to up-regulate cytokine gene expression [26]. The concentration of 2 cytokines was greater in the BAL of COPD mice exposed to CS + AFsp: TNF-α, a pro-inflammatory cytokine and potent neutrophil recruiter [22], as well as IL-10, a modulator of neutrophilic influx in the airways and of TNF-α expression [27]. CS exposure induces important alterations of gene expression via its effect on chromatin remodeling and DNA methylation [28], and also acts as a “double-edged sword” that either exacerbates pathological immune responses and dampens the defensive function of the immune system [29]. Although COPD mice have an altered gene expression profile, in the exacerbation model mediated by AFsp, we observed greater cytokine concentration in BAL of these mice that were related to structural tissue changes; in histopathological analyses, inflammation was found in mice combined with greater macrophage count in BAL following AFsp exposure. This inflammation was also reported in the analyses of gene expression in the lung, compared to control mice (induction of *IL-1α*, *IL-6*, *CCL-3* and *CCL-4*). At the protein level, the CS + AFsp group exhibited a strong inflammatory response compared to control mice (greater TNF-α, CXCL-1, IL-6, IL-4, IL-5, IL-10, IL-12(p70), G-CSF, CCL-3, CCL-4 and CXCL-2 concentration in BAL, and greater eotaxin concentration in serum). IL-4, IL-5, CCL-3, CCL-4 and eotaxin are involved in the chemotaxis of eosinophils. In addition, IL-4 plays a critical role in inflammation and pulmonary eosinophilia induced by *A. fumigatus* [30–34]. CXCL-1, CXCL-2, TNF-α, CCL-3 and CCL-4 are involved in anti-*A. fumigatus* response [35–37] and COPD [38–40]. It has previously been demonstrated that, when G-CSF, which showed the second highest elevated level in BAL fluids of CS + AFsp mice in the present study, is deleted, mice show lower lung inflammation and destruction; moreover, in BAL fluids of COPD patients, G-CSF is elevated [41]. Herein, induction of *CXCL-2*, *IL-6* and *CCL-4* gene expressions and, at the protein level, of only CXCL-2 were evidenced for CS + AFsp-treated mice when compared with CS-treated mice. Of note, 3 mice in the CS + AFsp group died during the smoking period, leaving only 5 for statistical analyses, which could limit the statistical power of the experiment. Another aspect that merits consideration is the “whole-body” smoke exposure of mice in the smoking chambers. A potential limitation of this method is that animals retain smoke molecules on their fur and thus ingest them when washing, which might influence the results [42].

It must be emphasized that we focused our study on inflammation, which is one mechanism that contributes to tissue destruction among other interconnected mechanisms such as protease-anti-protease imbalance and oxidative stress.

To our knowledge, studies exploring the immune response to *Aspergillus* in COPD are limited. In the study reported by Zhang et al. [43], the authors found that HMGB1 was responsible for the inflammatory response induced by *A. fumigatus* in COPD alveolar macrophages; in this study, TNF-α, IL-1β, IL-6 and IL-33 were significantly increased in COPD + AF compared to COPD. These results, together with those presented herein, suggest a worsening of inflammatory response in COPD after exposure to AFsp. However, these inflammatory changes caused tissue damage at alveolar level with release of cytokines (cytokines which are upregulated and others downregulated) from inflammatory cells that were recruited. These cells will also release proteases (e.g. elastase from neutrophils) and oxidative systems, which contribute to disruption of alveolar wall, hence tissue destruction. And, this means increasing the severity of COPD and symptoms (e.g., hypoxia) all which leading to poor disease prognosis. Moreover, the concentration of cytokines, such as IL-4, IL-5, IL-6, IL-10, IL-12(p70) and TNF-α, that we found to be higher in CS + AFsp-treated mice compared to controls, are involved in the differentiation of Th1/Th2 T cells and M1/M2 macrophages. In this way, we hypothesize that the worsening of the COPD symptoms might be due to the exacerbation of the inflammation, in particular, the change in the phenotypes of lymphocytes and/or macrophages.

## Conclusions

The data from both cell culture models and mice models bring new insights as to the pathophysiology of COPD exacerbations. They suggest a role of *Aspergillus fumigatus* in lung inflammation-mediated COPD exacerbation. These findings should stem further studies about the inflammatory cascade induced by AFsp in CS exposure models and how they relate to other well-documented mechanisms of COPD pathogenesis such as proteases- anti-proteases imbalance and oxidative stress.

## Electronic supplementary material

Below is the link to the electronic supplementary material.

Supplementary Material 1

## Data Availability

The data will be supplied upon request to corresponding author.

## Abbreviations

ABPA: Allergic bronchopulmonary aspergillosis
AFsp: *Aspergillus fumigatus* spores
ANOVA: Analysis of variance
BAL: Bronchoalveolar lavage
COPD: Chronic obstructive pulmonary disease
CS: Cigarette smoke
CSE: Cigarette smoke extract
Ct: Threshold cycle
DMEM: Dulbecco’s modified Eagle medium
ELISA: Enzyme-linked immunosorbent assay
FBS: Fetal bovine serum
HES: Hematoxylin-eosin-saffron
IPA: Invasive pulmonary aspergillosis
IQR: Interquartile range
SOPF: Specific and opportunistic pathogen-free
SD: Standard deviation
