# Supplementary material for: Aspergillus fumigatus is responsible for inflammation in a murine model of chronic obstructive pulmonary disease exacerbation
Source: Respir Res. 2025 Jan 18;26:25. doi: 10.1186/s12931-024-03092-7 (PMC11743040; doi:10.1186/s12931-024-03092-7)
Supplement: Supplementary file 1 — Supplementary Material 1 [file 12931_2024_3092_MOESM1_ESM.docx]

**SUPPLEMENTARY MATERIAL:**

**Supplementary Table S1:** Summary of pathological findings in formalin-fixed paraffin-embedded lung tissues

| Groups | Presence of inflammation and location | Type of inflammatory infiltrate | Inflammation intensity | Perivascular edema |
| --- | --- | --- | --- | --- |
| Control group (7 mice) | 14.3% (1/7)  perivascular inflammation and 14.3% (1/7)  bronchiolitis | Lymphocytes and plasma-cells (1/7) | - Mild: 14.3% (1/7)  - Moderate: 0%  - Severe: 0% | Absence |
| CS group  (7 mice) | 14.3 % (1/7) perivascular inflammation and 85.7 % (6/7) bronchiolitis | Lymphocytes and plasma-cells (1/7) | - Mild: 14.3% (1/7)  - Moderate: 0%  - Severe: 0% | 85.7% (6/7) |
| CS + AFsp group  (5 mice) | 100% (5/5)  perivascular inflammation, bronchiolitis and alveolitis | Lymphocytes and plasma-cells (5/5), rare neutrophils/eosinophils (4/5) and macrophages (1/5) | - Mild: 60% (3/5)  - Moderate: 40% (2/5)  - Severe: 0% | 100% (5/5) |

**Supplementary Table S2:** Assessment of the secretion of inflammatory mediators in BAL

|  | CS+AFsp vs controls | |
| --- | --- | --- |
| Inflammatory mediators | Fold increase | p-value |
| TNF-α | 1.3 | 0.0468 |
| CXCL-1 | 1.8 | 0.0403 |
| IL-6 | 4.5 | 0.0085 |
| IL-4 | 4.3 | 0.006 |
| IL-5 | 4.1 | 0.0029 |
| IL-10 | 2.1 | 0.0250 |
| IL-12(p70) | 4.8 | 0.03 |
| G-CSF | 13.8 | 0.0029 |
| CCL-3 | 7 | 0.003 |
| CCL-4 | 31 | 0.0162 |
| CXCL-2 | 1.7 | 0.0034 |

a)


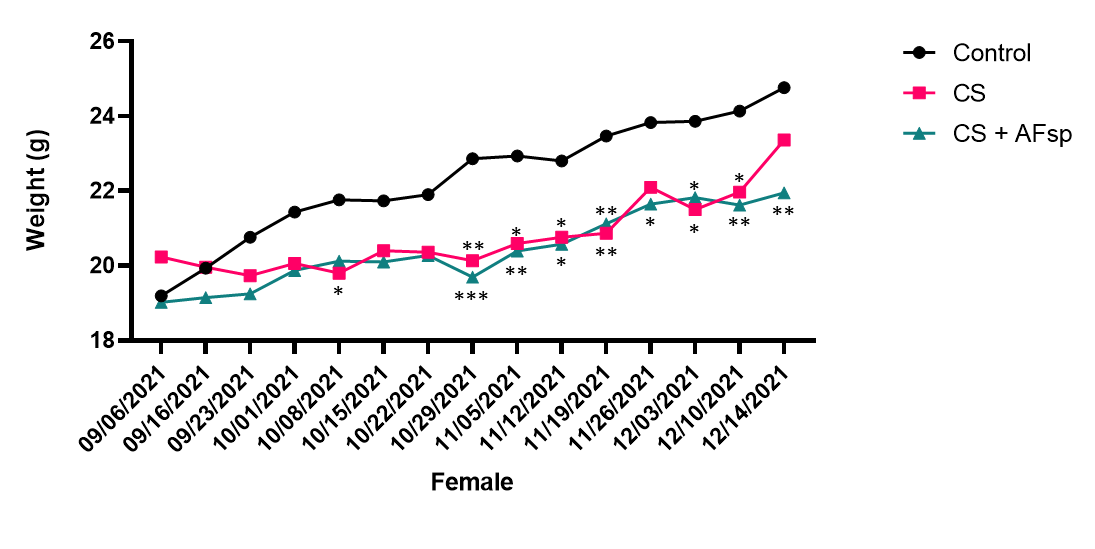


b)


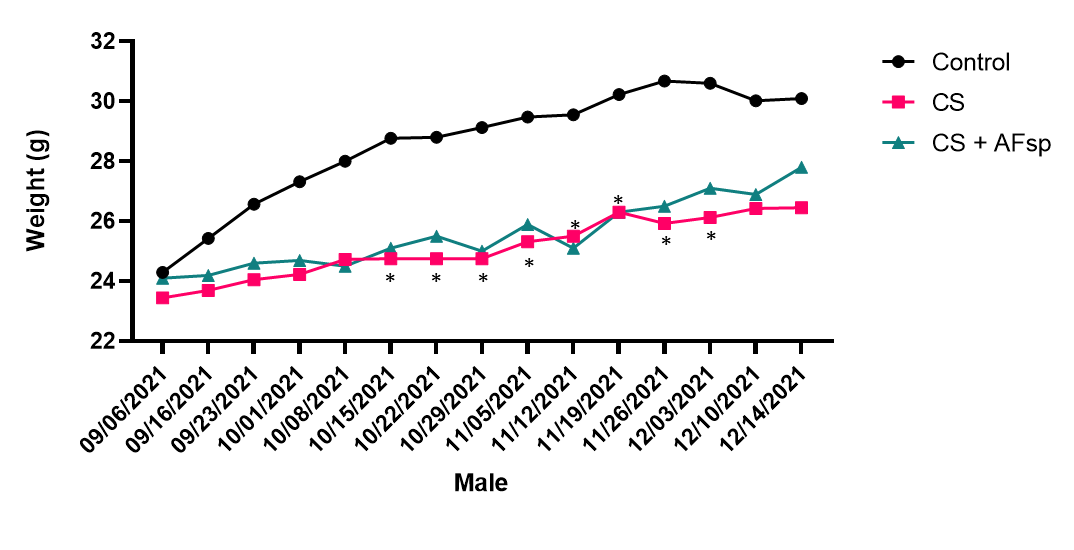


**Supplementary Figure S1: a) Weight of female and b) male mice after exposure to cigarette smoke (CS) and *A. fumigatus* spores (CS+AFsp).** Data are represented by mean at each time point (1 per week) for each group, n=5-7 mice per group, **: p<0.05, **: p<0.01.* No statistic has been performed for the CS+AFsp male group: only 1 male remained.
